# Mosquito (Diptera: Culicidae) diversity and medical importance in Koh Kong mangrove forests, Cambodia

**DOI:** 10.2478/abm-2022-0015

**Published:** 2022-06-30

**Authors:** Pierre-Olivier Maquart, Chea Sokha, Sébastien Boyer

**Affiliations:** Medical and Veterinary Entomology Unit, Institut Pasteur du Cambodge, Phnom Penh 12201, Cambodia; Wildlife Health Program, Wildlife Conservation Society, Sangkat Tonle Bassac, Phnom Penh 12000, Cambodia

**Keywords:** checklist, disease transmission, infectious, entomology, mosquito vectors

## Abstract

**Background:**

Mangroves are an ecosystem interface between land and sea, forming distinctive shallow-water marine communities in tropical and subtropical waters. The mangrove forest surface in Cambodia is being reduced due to deforestation. Because the mangrove type of ecosystem generally hosts a great diversity of mosquitoes, the urbanization of these ecosystems will increase interactions between humans and wild mosquitoes, and might thus serve as a potential source of new infectious diseases. Understanding mosquito diversity and analyzing their virome is critical to estimate the risk of emergence or future outbreaks of mosquito-borne diseases.

**Objective:**

To understand the mosquito diversity of mangrove forests of Koh Kong province (Cambodia).

**Methods:**

In 2019, the mosquito fauna was sampled for 3 consecutive days using BG-Sentinel and light traps, in 3 locations in the mangrove forests of Koh Kong province (Cambodia) during both dry and rainy seasons.

**Results:**

A total of 3107 samples were collected, belonging to 10 genera for 34 species. The *Culex* genus was the most diverse, accounting for 10 species. One species, *Culex sitiens,* represented over 60% of all collected mosquitoes. A total of 12 medically important species were recorded, 2 species, *Aedes* (*Stegomyia*) *albopictus* and *Culex vishnui*, were collected in all sites and during both the dry and rainy seasons, highlighting a potential risk of these species acting as bridge vectors.

**Conclusions:**

If new arboviruses were to be recorded in this peculiar area, it would indicate that the mosquito species found have the potential to act as a bridge between sylvatic and anthropogenic arboviruses.

Mangroves are a dominant ecosystem interface between land and sea [1], forming distinctive shallow-water marine communities in tropical and subtropical waters. They provide various ecosystemic functions, such as coastal protection against cyclones and tsunamis, protection of the shoreline, and carbon sequestration [2]. Due to the lengthy coastline and wide range of climate conditions, mangrove forests cover large areas of Asian coastlines [2]. Cambodia hosts the tenth largest mangrove ecosystem in Asia [3]. Its mangrove areas are distributed within 4 provinces: 79.8% in Koh Kong province (41,122 ha), 14.6% in Sihanouk province (7539 ha), 4.7% in Kampot province (2444 ha), and 0.9% in Kep province (498 ha) [3, 4]. Between 1989 and 2017, about 42% (36,810 ha) of the mangrove forests in Cambodia were cleared [3]. Despite a recently slowing pace [3], clearing continues, and costal anthropization in the region is booming.

Typically, mangrove forests, despite having a low plant diversity, are insect-species rich, having a very adapted fauna [5]. Notably, these areas, through the diversity of the aquatic habitat they provide, are well-known for their concentration of mosquitoes [6, 7, 8, 9]. These hematophagous insects have been found to form a great part of the mangrove entomological community, and in Southeast Asia are found to be particularly diversified in Malaysia, Thailand, Sumatra, and the Philippines [10].

Urbanization of mangrove forests increases potential interactions between humans, wild mosquitoes, and potentially new infectious diseases [11, 12]. Continuous ecological change, including fragmentation of natural ecosystems, may increase the probability of human–vector contact, and, consequently, the possibility of outbreaks of emerging and re-emerging arboviruses [13]. Previous studies on the virome of saltmarsh mosquito demonstrated the presence of several human-pathogenic viruses from the flavivirus and alphavirus families [11, 14] and their risk of emergence. Given the increasing focus on mangrove conservation across Southeast Asia [15], understanding the mosquito diversity, and subsequently analyzing their virome, is critical in being able to estimate the risks of the emergence and outbreaks of mosquito-borne diseases [14]. However, to our knowledge, there are no previous studies on the composition of the Culicidae fauna in the Cambodian mangroves. To our knowledge, the present article reports for the first time the mosquito fauna of the mangrove forests in Koh Kong province, providing a baseline of their biodiversity in southwestern Cambodia.

## Methods

### Collecting permit

This research was approved by Cambodian government authority and allowed by the Ministry of Environment (permit No. 1443, issued on November 15, 2018), and by the Directorate General of Nature Conservation and Protection (permit No. 057, issued on February 14, 2019).

### Study sites

**Figure 1:**
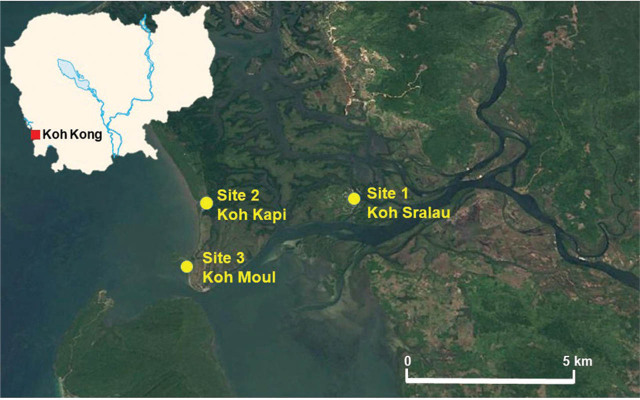
Location of the 3 different sampling sites on Koh Kong. The inset in white represents Cambodia, showing the general location of Kho Kong (red square). Site 1: Koh Sralau island, 1.43 km SSW of Sralau village (11°4502N, 103°0645E, WGS84). Site 2: Koh Kapi, 1 km E of the city (11°4544N, 103°0207E, WGS84). Site 3: Koh Moul island, 2.5 km S and 7.2 km SW of Koh Kapi and Koh Sralau, respectively (11°4329N, 103°0063E, WGS84), and a beach nearby (11°4306N, 103°0129E, WGS84). Modified from Google Maps version 10.66.1 (imagery date November 1, 2019); imagery and data attributions: imagery CNES/Airbus, Landsat/Copernicus, Maxar Technologies, ©2022 TerraMetrics; map data SIO, NOAA, U.S. Navy, NGA, GEBCO ©2022 Google.

For the field survey, 3 main sites were investigated. The first one was on Koh Sralau island, located 1.43 km SSW of Sralau village (11°4502N, 103°0645E, World Geodetic System (WGS) 84, geographical coordinates in decimal degrees). There, 6 U.S. Centers for Disease Control and Prevention (CDC) Mini Light Traps and 6 BG-Sentinel 1 traps (Biogents) were placed in the mangrove forest for 3 consecutive days (**Figure 1**). The second site was on Koh Kapi and was investigated using 10 traps (5 CDC Mini Light Traps and 5 BG-1 traps) placed in the mangrove forest for 3 consecutive days, 1 km E of the city (11°4544N, 103°0207E, WGS84) (**Figure 1**). The third sampling site was on Koh Moul Island, where 1 CDC Mini Light Trap and 1 BG-1 trap were placed on the island itself, 2.5 km S and 7.2 km SW of Koh Kapi and Koh Sralau, respectively (11°4329N, 103°0063E, WGS84), while 2 CDC Mini Light Traps and 2 BG-1 traps were set up in the beach nearby (11°4306N, 103°0129E, WGS84), for 3 consecutive days (**Figure 1**).

### Collection methods

Adult mosquitoes were collected using BG-Sentinel 1 Mosquito Traps, 7.5–12 V, coupled with BG-Lure (BioQuip) and CDC Mini Light Traps (BioQuip), baited with incandescent light, with dry ice (solid CO_2_, which sublimes to release CO_2_ gas) placed in a dispenser next to the traps. Mosquitoes were killed humanely using carbon dioxide. Each trapping location was visited daily to remove the collected insects.

**Figure 2:**
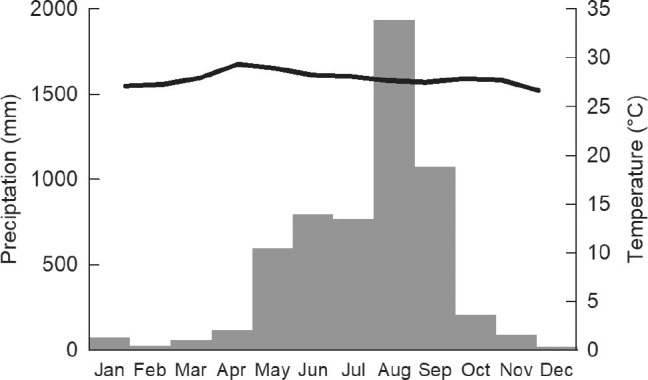
Meteorological conditions in Koh Kong province in 2019. Climate data were obtained from climateengine.org. Temperatures (solid line) were gathered from Terraclimate and CFSR satellite data, and precipitation (gray bars) from CHIRPS satellite data.

The fieldwork was carried from at the end of March 2019, for the dry season, and in mid-November 2019 for the rainy season. The meteorological conditions observed in 2019 did not differ from those of the previous years. Climate data were obtained from climateengine.org (**Figure 2**). The temperatures were gathered using Terraclimate and CFSR satellite data, while the precipitation was obtained from CHIRPS satellite data.

### Morphological identification

The mosquitoes were kept individually in Eppendorf tubes, identified on the field, and kept in an electric tank cooler at 4°C for the time of the mission. They were identified using their diagnostic morphological characters [16, 17, 18, 19, 20, 21, 22, 23, 24, 25]. The identification was confirmed by the examination of the original description of the species. Voucher specimens are deposited in the collection of the Institut Pasteur du Cambodge, Phnom Penh, Cambodia.

## Results

In total 3107 mosquitoes were collected, of which 89.4% (2777 specimens) were identifiable to the species level (**Table 1**). During the study, 34 species from 10 genera were collected. The collection included 11 species of *Culex*, 5 *Aedes*, 4 *Mansonia*, 3 *Coquilletidia*, 3 *Mimomyia*, 3 *Uranotaenia*, 2 *Anopheles*, 1 *Armigeres*, 1 *Heizmannia*, and 1 species of *Verralina*.

**Table 1:** List and total number of mosquitoes species collected in Koh Kong province

**Mosquito species**	**Koh Sralau**		**Koh Kapi**		**Koh Moul**		**Total**
		
**Season**	**Dry**	**Rainy**	**Dry**	**Rainy**	**Dry**	**Rainy**	
Aedes (5 species)							141
*Aedes* (*Stegomyia*) *aegypti* (Linnaeus, 1762)		9			1		10
*Aedes* (*Stegomyia*) *albopictus* (Skuse, 1894)	1	6	1	3	1	2	14
*Aedes* (*Lorrainea*) amesii (Ludlow, 1903)		4					4
*Aedes* (*Rhinoskusea*) *longirostris* (Leicester, 1908)				30			30
*Aedes* (*Phagomyia*) *prominens* (Barraud, 1923)		1					1
*Aedes* sp.	8	41	1	31		1	82

*Anopheles (2 species)*							*42*

*Anopheles* (*Anopheles*) *baezai* Gater, 1934	2	4			1		7
*Anopheles* (*Cellia*) *indefinitus* Ludlow, 1904	1						1
*Anopheles* sp.	13	20				1	34

*Armigeres* (1 species)							5

*Armigeres* (*Armigeres*) *subalbatus* (Coquillett, 1898)	1	1		1			3
*Armigeres* sp.	1	1					2

*Coquilletidia* (3 species)							66

*Coquillettidia* (*Coquillettidia*) *crassipes* (van der Wulp, 1881)	1	1		9			11
*Coquillettidia* (*Coquillettidia*) *nigrosignata* (Edwards, 1917)		51					51
*Coquillettidia* (*Coquillettidia*) *ochracea* (Theobald, 1903)		2		2			4

*Culex* (11 species)							2571

*Culex (Oculeomyia) bitaeniorhynchus* Giles, 1901	*1*	*13*					*14*
*Culex (Eumelanomyia) brevipalpis* (Giles, 1902)				*1*		*1*	*2*
*Culex* (*Culex*) *fuscocephala* Theobald, 1907		21		1			22
*Culex* (*Culex*) *gelidus* Theobald, 1901		6		1			7
*Culex* (*Eumelanomyia*) *malayi* (Leicester, 1908)		1					1
*Culex* (*Culex*) *quinquefasciatus* Say, 1823	1						1
*Culex* (*Lophoceraomyia*) *reidi* Colless, 1965		6					6
*Culex* (*Culex*) *sitiens* Wiedemann, 1828		682		1103		89	1874
*Culex* (*Culex*) *tritaeniorhynchus* Giles, 1901	3						3
*Culex* (*Culex*) *vishnui* Theobald, 1901	27	120	230	85	8	2	472
*Culex* (*Lophoceraomyia*) *wilfredi* Colless, 1965		37		7			44
*Culex* sp.	8	109	1	3	4		125

*Heizmannia* (1 species)							1

*Heizmannia* sp.		1					1

*Mansonia* (4 species)						120	

*Mansonia* (*Mansonioides*) *bonneae* Edwards, 1930		81		1			82
*Mansonia* (*Mansonioides*) *dives* (Schiner, 1868)	2	11					13
*Mansonia* (*Mansonioides*) *indiana* Edwards, 1930		6					6

*Mansonia* (*Mansonioides*) *uniformis* (Theobald, 1901)	2						2
Mansonia sp.	2	15					17

*Mimomyia* (3 species)							8

*Mimomyia* (*Etorleptiomyia*) *elegans* (Taylor, 1914)		1					1
*Mimomyia* (*Mimomyia*) *hybrida* (Leicester, 1908)	1	3					4
*Mimomyia* (*Etorleptiomyia*) *luzonensis* (Ludlow, 1905)		3					3

*Uranotaenia* (3 species)							110

*Uranotaenia* (Pseudoficalbia) *hirsutifemora* Peters, 1964		6					6
*Uranotaenia* (*Uranotaenia*) *metatarsata* Edwards, 1914		1					1
*Uranotaenia* (*Uranotaenia*) *rampae* Peyton & Klein, 1970		9		53		9	71
*Uranotaenia* sp.	1	20	1	2	4	4	32

*Verrallina* (1 species)							43

*Verrallina* (*Verrallina*) *dux* (Dyar & Shannon, 1925)		5					5
*Verrallina* sp.		14				6	20

Unknown	13	2	3			18	

Grand total	89	1314	237	1333	19	115	3107

Number of species	12	28	2	13	4	6	34
Number of species per collection site	32		13		7		
Shannon index	1.7	1.5	0.09	0.06	0.9	0.7	
Shannon index	1.6		0.9		0.9		
Simpson index	0.7	0.6	0.03	0.3	0.4	0.3	
Simpson index	0.6		0.4		0.4		

The genus *Culex*, besides being the most diversified, was also the most abundant with 2571 specimens, representing 82.75% of all collected mosquitoes. One species in particular, *Culex sitiens*, was over-dominant (n = 1874) representing 60% of all collections. This species was present in all sites studied, but occurred only during the rainy season. The second most abundant species, *Culex vishnui*, representing 15% of all collected species (n = 472 specimens), was also present in all the sites, but was found throughout the year.

The core of the reserve, Koh Sralau, located in the mangrove forest, harbored the highest Culicidae diversity with 32 species (**Table 1**). The lowest diversity was observed in Koh Moul, the site located near the beach, with only 7 species (**Table 1**). The diversity and number of collected mosquitoes were the highest during the rainy season: during the dry season, only 14 species and 345 specimens were collected, whereas 29 species and 2762 specimens were observed during the rainy season (**Table 1**).

Only 8 species could be found through the year: *Aedes albopictus, Anopheles baezai, Armigeres subalbatus, Coquillettidia crassipes, Culex bitaeniorhynchus, Cx. vishnui, Mansonia dives*, and *Mimomyia hybrida.* Except the last 2, all of these species are considered to be of medical importance.

During our collection, a total of 12 species from 5 different genera are reported to be of medical importance (**Table 2**) and represented 77.9% (n = 2423) of all collected mosquitoes.

**Table 2:** List of medically important mosquito species in Koh Kong

**Mosquito species**	**Potential vectors**	**References**
*Aedes (Stegomyia*) *aegypti* (Linnaeus, 1762)	DENV, ZIKV, CHYKV, WNV, YFV	[26, 27, 28]
*Aedes (Stegomyia*) *albopictus* (Skuse, 1894)	DENV, ZIKV, CHYKV, JEV, WNV	[26, 27, 28]

*Anopheles* (*Cellia*) *indefinitus* Ludlow 1904	MAL	[26, 29]

*Armigeres* (*Armigeres*) *subalbatus* (Coquillett, 1898)	JEV, FILWB	[26, 27, 30]

*Culex* (*Oculeomyia*) *bitaeniorhynchus* Giles, 1901	JEV, FILBM, FILWB	[26, 30]
*Culex* (*Culex*) *fuscocephala* Theobald, 1907	JEV	[26, 30]
*Culex* (*Culex*) *gelidus* Theobald, 1901	JEV	[26, 27, 30]
*Culex* (*Culex*) *quinquefasciatus* Say, 1823	ZIKV, JEV, WNV	[26, 27, 30]
*Culex* (*Culex*) *sitiens* Wiedemann, 1828	FILBM, JEV	[26, 30, 31, 32]
*Culex* (*Culex*) *tritaeniorhynchus* Giles, 1901	JEV	[26, 27, 30]
*Culex* (*Culex*) *vishnui* Theobald, 1901	JEV	[26, 27, 30]

*Mansonia* (*Mansonioides*) *uniformis* (Theobald, 1901)	WNV	[26]

CHIKV, chikungunya virus; DENV, dengue virus; FILBM, filariosis by *Brugia malayi*; FILWB, filariosis by *Wucheria bancrofti*; JEV, Japanese encephalitis virus; MAL, human *Plasmodium spp*.; WNV, West Nile virus; YFV, yellow fever virus; ZIKV, zika virus.

## Discussion

The present study provides—to our knowledge—the first list of mosquitoes in the mangrove forests of Koh Kong. Besides providing a baseline for future researches in this conserved area, it is another step forward in assessing the local risks of mosquito-borne disease.

Of the 290 known species recorded from Cambodia [26], the mosquitoes sampled in Koh Kong, with 37 recorded species, represent 12.7% of the Cambodian fauna. The Culicidae fauna of Koh Kong is quite diverse compared with that in the mangrove forest at the mouth of the Bangpakong river in Thailand, where only 14 species were recorded [33]. The most extensive work in the southern Asian region has been conducted in the Indian mangrove forests, where 43 species were recorded in Bhitarkanika (Orissa; [6]), 19 in the Sunderbans (West Bengal; [7]), 53 in the Andaman Islands [8], and 12 species in Coringa (Andhra Pradesh; [9]). Typically, mangrove forests, despite having a low plant diversity, are insect-species rich, having a very adapted fauna [5].

Most of the species collected belonged to the genus *Culex,* where 11 species were recorded. One species in particular, *Cx. sitiens*, is over-dominant during the rainy season, representing 60.3% of all collected mosquitoes (1874 specimens collected), and was collected across all sites. This result is not surprising because *C. sitiens* is strongly associated with mangrove forests [31], and is considered to be a saltmarsh mosquito [34, 35]. Easton [35] has made similar observations in mangroves in Macau, where this species was the second-most abundant, after *Cx. quinquefasciatus.* The latter species was only collected once in Koh Sralau during the rainy season. According to T Ismail et al. [15], *Cx. sitiens* was found to be predominant in the mangrove forest of Merbok (Malaysia), but in the present study, only after *Verralina butleri*, a species that was not observed in Koh Kong. The seasonality seems important for the members of the *Culex* genus, where only 6 species were collected during the rainy season.

In total, 5 species of *Aedes* were collected. All were collected (except *Ae. albopictus*) during the rainy season. Interestingly, despite a 5-year extensive collection through 15 provinces in Cambodia and the collection of more than 230,000 mosquitoes [26], *Aedes* (*Lorrainea*) *amesii* (n = 4) and *Aedes* (*Rhinoskusea*) *longirostris* (n = 30) were collected only in the Koh Kong mangrove forests. Both species are attracted to mangrove swamps [23, 36]. Immature stages of the subgenus *Rhinoskusea* are usually confined to brackish water coastal and river areas, and can be found in crab holes and small ground pools associated with mangroves [37]. The 2 *Stegomyia* species, *Ae. albopictus* and *Ae. aegypti*, have been reported from mangrove areas, being able to develop in brackish waters [38]. For the last *Aedes* species, *Ae.* (*Phagomyia*) *prominens*, very little is known about its biology [37], but this species was previously recorded in Cambodia in Mondulkiri province [26], suggesting that its distribution is not exclusive to mangrove ecosystems.

The genus *Anopheles* is poorly represented, with only 2 species recorded. According to Klein [39], *An. baezai* and *An. indefinitus* (named *An. sundaicus* in his article) are typical coastal *Anopheles* breeding in brackish waters on coastal regions. They are usually restricted to a narrow fringe along the coastline [39]. These data are consistent with our observations, where only 8 specimens were collected, mainly on Koh Sralau island and on the beach nearby.

The genera *Mimomyia* and *Uranotaenia* were present with 3 species for each genus. Both genera are typical saltmarsh mosquitoes. While very little is known about the biology of *Mimomyia*, the immature stages occur in swamps and marshes with dense vegetation. The adults of several species have been reported to bite humans, but none are considered to be serious pests [20]. Species of the genus *Uranotaenia* are known to bite mainly amphibians and reptiles, but birds and mammals can serve as secondary hosts. This genus is not known to be involved in any pathogen transmission [20]. The immature stages of *Uranotaenia* species utilize a wide range of habitats, including swamps and salt marshes [37].

*Verrallina dux* were collected only in Koh Sralau (the core of the reserve), and while the adults can be found resting in crab holes, its larvae can be found in temporary stagnant water holes, such as in footprints in rice fields, and are reported to be common in mangrove swamps in salt marshes and brackish waters [40]. *Mansonia* larvae occur generally in permanent waters and are associated with aquatic plants such as water lettuces (*Pistia sp.*). The larvae attach themselves to the floating roots of the plant to obtain oxygen from their aeriferous parenchyma. They can detach from and reattach to their host plants quite readily. Species of the genus *Mansonia* display the same biology and are also common in mangrove forests [35]. Females of several species in both genera are nocturnal biters, known to feed on humans and involved in the transmission of various arboviruses [37].

In total, 12 species of medical importance belonging to 5 different genera were collected, and represented 77.9% (n = 2423) of all collected mosquitoes. Most were collected during the rainy season.

Some 9 different species are vectors of Japanese encephalitis virus (JEV). The 2 main JEV vector species recognized are *Culex tritaeniorhynchus* and *Cx. gelidus* [30], both present in mangrove forests. The remaining 7 species are confirmed vectors: *Ae. albopictus, Ar. subalbatus, Cx. fuscocephala*, *Cx. bitaeniorhynchus*, *Cx. sitiens*, *Cx. quinquefasciatus*, and *Cx. vishnui* [30]. The JEV epidemiological cycle is undertaken through several hosts: wild birds, pigs, and mosquitoes, and is considered to be the most important cause of human encephalitis in Southeast Asia, and well established in Cambodia [12, 27, 30].

The 2 *Stegomyia* species (*Ae. aegypti* and *Ae. albopictus*) present in Koh Kong are considered to be potential vectors of dengue, Zika (along with *Cx. quinquefasciatus*), and chikungunya viruses [28]. Even though there is no evidence of intense circulation of West Nile virus (WNV) in Cambodia [41], concerns are arising about the ability for WNV to spread along bird migration routes based on its recent expansion in Eurasia [41]. This virus is naturally maintained in an enzootic transmission cycle of mosquitoes acting as vectors, and birds acting as the amplifying hosts. Humans and other mammals can serve as accidental/dead end hosts [42]. In Koh Kong, 2 more species are known vectors, namely *Cx. quinquefasciatus* and *Mansonia uniformis* [43]. All of them are known to be zoo–anthropogenic species [44, 45]. Blood meal analysis of *Cx. quinquefasciatus* demonstrated that they are highly ornithophilic besides feeding on mammals [46]; therefore, it is most likely that this vector can act as an important bridge vector and contribute to viral amplification [43].

Although only one specimen of parasite-carrying mosquitoes was captured, *Anopheles indefinitus* is reported to be potential vector of malaria [29, 47, 48, 49, 50] and 4 species are known to transmit filaria parasites, namely *Ar. subalbatus* and *Cx. bitaeniorrhynchus* transmitting *Wucheria bancrofti*, and *Cx. sitiens* and *Cx. bitaeniorrhynchus* transmitting *Brugia malayi* [49, 51]. However, these parasites were not yet recorded in Cambodia.

On a more general note, of the 12 medically important species, 3 of them were caught in each of the 3 sites. *Cx. sitiens*, a JEV vector, represents 60.3% of all collected mosquitoes and was found in all sites, but only during the rainy season. However, the 2 species *Ae. albopictus* and *Cx. vishnui* were found during both seasons. The first of these species is circulating at low intensity (representing 0.45% of all collected mosquitoes), but is a known vector of several arboviruses of high human medical importance [28]. The second species *Cx. vishnui* represents 15% of all collected mosquitoes in all habitats and almost all seasons. By contrast, in other sites in Cambodia, it usually accounts for more than half of the collected mosquitoes. Between 2017 and 2018, Boyer et al. [12] surveyed the mosquito population in 24 schools in Kampong Cham and Tboung Khmum provinces in Cambodia. Of 37,725 mosquitoes collected, 51.8% were *Cx. vishnui*. The over-dominance of this species was recorded in several other regions in Cambodia, such as in Prek Toal, where they represented 59.4% of all collected mosquitoes [52]. These 2 species, present throughout the sites, throughout the year, and across Cambodia, may represent a risk as bridge vectors for new or emergent zoonotic diseases. The rapid development of human settlements, and continued urbanization of ecosystems that are habitats for a rich diversity of wild fauna, represents a risk, as the dispersion of anthropophilic mosquito species into previously unfavorable habitats [53] could modify vector–host interactions. This could potentially lead to more contact with sylvatic reservoirs of zoonotic pathogens [54], and favors the emergence of new or neglected diseases in human populations.

Limitations of the present study include that each site was visited only once per season (rainy and dry) and that only BG and light-traps were used to collect mosquitoes. Increasing the diversity of traps and methods such as larvae collection are the best ways to improve the diversity of the collected Culicidae.

### Importance for public health

While the clearing of the Cambodian mangroves pace is slowing due to reforestation practices and proper law enforcement, further efforts are needed in this direction [3]. This coastal habitat could provide a buffering effect toward vector borne disease [11], and negatively affects arbovirus transmission [11]. While the conservation of biodiversity-rich areas is essential, with the striking resurgence of arboviruses, such as Zika, dengue, yellow fever, or chikungunya this century [55], the knowledge of blood-sucking arthropods—potentially vectors of diseases—and the pathogens they can carry is becoming increasingly necessary for public health considerations. In the present study, 2 species of medical importance were found throughout the year, and across all the different sites, namely *Ae. albopictus* and *Cx. vishnui*. Both species, very common in Cambodia, display a zoo–anthropogenic behavior, and are anthropophilous. If new arboviruses were to be recorded in this peculiar area, these species would have the potential to act as a bridge between sylvatic and anthropogenic arboviruses. Further studies should be conducted using next generation sequencing technology to investigate the virus diversity among these vectors in Koh Kong province to assess the risk of disease emergence.
